# Maternal Overnutrition During Gestation in Sheep Alters Autophagy Associated Pathways in Offspring Heart

**DOI:** 10.3389/fgene.2021.742704

**Published:** 2022-01-31

**Authors:** Yang Liu, Qiyue Ding, Steven J. Halderson, Sebastian I. Arriola Apelo, Amanda K. Jones, Sambhu M. Pillai, Maria L. Hoffman, Sarah Reed, Kristen E. Govoni, Steven A. Zinn, Wei Guo

**Affiliations:** ^1^ Department of Animal and Diary Sciences, University of Wisconsin-Madison, Madison, WI, United States; ^2^ Department of Animal Science, University of Connecticut, Storrs, CT, United States

**Keywords:** poor maternal nutrition, maternal obesity, developmental programming, autophagy, sheep, heart muscle

## Abstract

Poor maternal nutrition during gestation can negatively affect offspring growth, development, and health pre- and post-natally. Overfeeding during gestation or maternal obesity (MO) results in altered metabolism and imbalanced endocrine hormones in animals and humans which will have long-lasting and detrimental effects on offspring growth and health. In this study, we examined the effects of overnutrition during gestation on autophagy associated pathways in offspring heart muscles at two gestational and one early postnatal time point (*n* = 5 for treated and untreated male and female heart respectively at each time point). Two-way ANOVA was used to analyze the interaction between treatment and sex at each time point. Our results revealed significant interactions of maternal diet by developmental stages for offspring autophagy signaling. Overfeeding did not affect the autophagy signaling at mid-gestation day 90 (GD90) in both male and female offspring while the inflammatory cytokines were increased in GD90 MO male offsrping; however, overfeeding during gestation significantly increased autophagy signaling, but not inflammation level at a later developmental stage (GD135 and day 1 after birth) in both males and females. We also identified a sexual dimorphic response in which female progeny were more profoundly influenced by maternal diet than male progeny regardless of developmental stages. We also determined the cortisol concentrations in male and female hearts at three developmental stages. We did not observe cortisol changes between males and females or between overfeeding and control groups. Our exploratory studies imply that MO alters autophagy associated pathways in both male and female at later developmental stages with more profound effects in female. This finding need be confirmed with larger sample numbers in the future. Our results suggest that targeting on autophagy pathway could be a strategy for correction of adverse effects in offspring of over-fed ewes.

## Introduction

Developmental programming, also known as fetal programming, occurs during *in utero* life whilst the fetus is developing (Widdowson and McCance, 1975). During this specific window of development when the fetus is especially vulnerable, exposure of the fetus to an unfavorable uterine environment such as poor nutrition or hormonal perturbations may lead to retarded offspring growth and short- and long-term health implications (Barker et al., 1993; Barker, 2007; Gluckman et al., 2008). Nutrition is one of the major intrauterine environmental factors that can reprogram fetal growth and development during gestation in many species such as cattle, swine and sheep (Bell and Ehrhardt, 2002). Both maternal under- and over-nutrition can lead to intrauterine growth restriction, reduced birth weight, increased fetal and neonatal mortality, and altered postnatal growth rate, decreased carcass quality, feed efficiency, and negative health effects in animals and humans (Barker and Clark, 1997; Godfrey and Barker, 2001; Bell and Ehrhardt, 2002; Wallace et al., 2003; Wu et al., 2006; Ford and Long, 2011; Du et al., 2013; Reed et al., 2014).

Due to the health-associated risk factors, obesity has become a major health issue worldwide and is comorbid with increased global rates of a variety of chronic conditions including heart disease, diabetes, hypertension, elevated cholesterol, stroke, heart failure, cancers, and arthritis during early life and in adulthood (Malnick and Knobler, 2006). Further, the prevalence of maternal obesity (MO) in the United States is high and increasing (Ogden et al., 2007; Flegal et al., 2010). Nearly one-third of women are obese at child-bearing age (Caballero, 2003; Boney et al., 2005; Flegal et al., 2012; Zambrano and Nathanielsz, 2013). MO induces adverse effects on both maternal health and fetal growth and development, which can result in harmful, and persistent effects in offspring (Friedrich, 2002; Sullivan et al., 2011). Human epidemiological studies have shown that MO triggers cardiac remodeling and increases risks of offspring heart disease later in life (Reynolds et al., 2013; Gaillard, 2015). Using sheep as a model, our group revealed that MO impaired fetal cardiomyocyte contractile function by altering myofilament protein composition and disrupting calcium homeostasis through altered intracellular calcium handling signaling (Wang et al., 2019). Other research groups found that over-nutrition and MO alters the JUN N-terminal kinase (JNK)-insulin receptor substrate (IRS)-1 signaling cascade and cardiac function in the fetal heart (Wang et al., 2010) and induces fibrosis in fetal myocardium of sheep (Huang et al., 2010). Knowledge gained from the past decades has shown that energy imbalance and hormonal dysregulation are widely accepted etiological mechanisms underpinning obesity that are tightly regulated by autophagy (Zhang et al., 2018). In this study, we aimed at understanding whether MO-induced cardiac dysfunction is associated with altered autophagy signaling. We used a previously characterized MO sheep model (Pillai et al., 2017) to determine whether autophagy associated cell signaling pathways are changed in fetuses and neonates at different developmental stages in response to MO.

## Methods and Materials

### Animals

All animal procedures were reviewed and approved by the University of Connecticut Institutional Animal Care and Use Committee (A13-059). Animal procedures and complete experimental design details were described previously (Pillai et al., 2017). Briefly, multiparous Western White-faced ewes (*n* = 36) were estrus synchronized using a progesterone controlled intravaginal drug release device (Easi-Breed CIDR Sheep Insert, Zoetis Inc., Parsippany, NJ), followed by a single i. m. injection of prostaglandin F_2_ alpha (Lutalyse, 5 mg/ml; Zoetis, Inc.). Ewes were bred to 1 of 4 related Dorset rams. A rump mark received by the ewe was considered as Day 0 of pregnancy. After 20 days, ewes were then housed in individual pens. Pregnant ewes at gestation day 30.2 ± 0.2 were fed either a control (100% NRC; CON; *n* = 17) or over-fed diet (140% NRC; MO; *n* = 19) based on National Research Council (NRC) requirements for total digestible nutrients (TDN, National Research Council, 1985). Ewes at gestation day 90 (*n* = 6 CON, 6 MO) or 135 (*n* = 6 CON; 7 MO) were euthanized by an intravenous injection of Beuthanasia-D Special (Merck Animal Health; Summit, NJ) containing 390 mg/ml sodium pentobarbital and 50 mg/ml phenytoin based on body weight, and exsanguinated. A hysterectomy was performed to remove the uterus and all fetuses for fetal sample collection. A subset of ewes (*n* = 5 CON; 6 MO) were allowed to give birth. Lambs were nursed for up to 24 h, weighed, euthanized with an i., v. overdose of Beuthanasia-D Special (390 ng/ml sodium pentobarbital and 50 mg/ml phenytoin based on body weight), and exsanguinated (Pillai et al., 2017; Martin et al., 2019; Gauvin et al., 2020).

### Sample Collection

To obtain fetal organs, a mid-ventral incision extending from the thoracic cavity to the lower abdominal cavity was made. Heart was excised from each offspring (*n* = 5 each from control and treatment group at different developmental stages). The heart was weighed and heart length and width were measured. Then heart tissues were snap-frozen in liquid nitrogen and stored at −80°C freezer until analyzed.

### Protein Sample Preparations

Ventricles of offspring sheep heart were weighed and approximately 50 mg was homogenized with a glass tissue grinder in 1 ml of Urea—Thiourea Sample Buffer [8 M urea, 2 M thiourea, 75 mM DTT, 3% SDS, 0.05% bromophenol blue, and 0.05 M Tris (pH6.8)] as described previously (Guo et al., 2012; Wang et al., 2019). After samples were completely dissolved, the protein samples were transferred to a 1.5 ml centrifugation tube, and heated to 60°C for 10 min. The protein samples were then centrifuged at 16,000 rpm for 10 min at 4°C. The supernatants were transferred to a new 1.5 ml centrifugation tube and aliquoted and stored at −80°C for later analysis.

### Western Blotting

Total protein was separated by SDS-PAGE for 1.5 h with 120 voltage and transferred onto a PVDF membrane (Bio-Rad, Hercules, CA, Catalog #1620177) using Bio-Rad Trans-Blot® Turbo™ Transfer System (7 min with standard protocol provided by the manufacturer). The membrane was blocked with 5% non-fat milk for 2 hours at room temperature (RT) and probed overnight at 4°C with the following antibodies with dilution range from 1:500 to 1,500: LC3 rabbit antibody (1:1,000) (Catalog # 2775S; Cell Signaling), SAPK/JNK rabbit antibody (1:500) (Catalog # 9252S; Cell Signaling), anti-rabbit Phospho-SAPK/JNK rabbit antibody (1:1,000) (Catalog # 4,668; Cell Signaling), phospho-AKT473 rabbit antibody (1:1,500) (Catalog # 4060S; Cell Signaling), P38 MAPK rabbit antibody (1:1,000) (Catalog # 8690S; Cell Signaling), phosphor-p38 MAPK rabbit antibody (1:1,000) (Catalog # MA515182; Invitrogen), NK-κB rabbit antibody (1:1,000) (Catalog # 50–172–9,292; Proteintech), TNFα rabbit antibody (1:1,000) (Catalog # PBOTNFAI; Invitrogen), Atg5 rabbit antibody (1:1,500) (Catalog # 12994S; Cell Signaling), AKT (pan) rabbit antibody (1:1,000) (Catalog # 4691S; Cell Signaling), Rab7 rabbit antibody (1:1,500) (Catalog # 9367S; Cell Signaling), anti-GAPDH rabbit mAB (1:1,000) (Catalog # 2118S; Cell Signaling), p44/42 MAPK Horseradish Peroxidase (HRP) conjugated rabbit antibody (1:500) (Catalog # 4348S; Cell Signaling), phosphor-p44/42 MAPK HRP conjugated rabbit antibody (1:1,000) (Catalog # 8544S; Cell Signaling). HRP conjugated secondary antibodies were then incubated with anti-rabbit IgG (1:5,000) (Catalog # 4,011; Promega corporation) for 1 hour at RT. Membranes were developed using the SuperSignal West Pico PLUS Chemiluminescent Substrate (Catalog # 34,579; Thermo Scientific) and signals were obtained using ChemiDoc MP imaging system (Bio-Rad, Hercules, CA). The detailed procedure is found in our previous publication (Wang et al., 2019).

### Heart Tissue Cortisol Level Measurement

Fifty mg of heart tissue from each group (*n* = 5) were lysed in PBS through homogenization with a Dounce homogenizer. The lysate was diluted 1:10 in diethyl ether and vortexed. The organic layer was recovered, dried under N gas and resuspended in cortisol ELISA buffer (Arbor Assays, #K003). Cortisol was measured by ELISA following provider instructions (Arbor Assays, Ann Arbor, and MI) and previous publication (Stillo et al., 2021).

### Statistical Analysis

Prism software (GraphPad, La Jolla, CA) was used for statistical analysis. Results were expressed as means ± SEM. Statistical significance was determined with two-way ANOVA analysis of differences affected by two factors: treatment and sex at each time point. Bonferroni’s multiple comparisons test were used to determine the significant differences between each type of treatment within same gender, or each gender within same treatment. Significance was set at values of *p* < 0.05.

## Results

### Activation of MAPK Signaling in Fetal and Neonatal Sheep Hearts of Offspring of Maternal Obese Ewes

**FIGURE 1:**
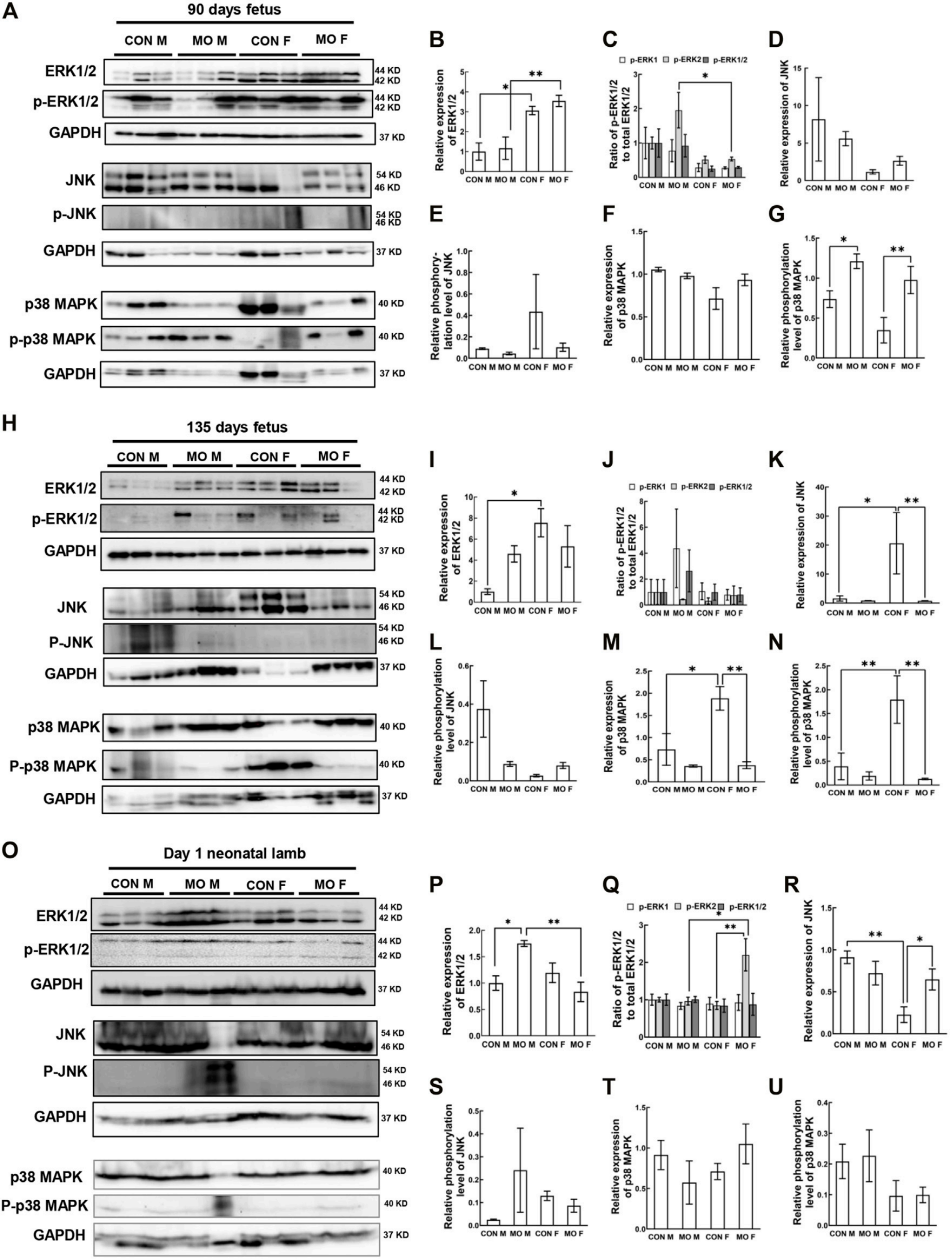
Alteration of MAPK/ERK signaling pathway in offspring heart of overfed ewes at two gestational and one early postnatal time point. **(A–G)**. Relative expression and phosphorylation level of hallmark proteins in fetal heart at mid-gestation day 90; **(H–N)**. Relative expression and phosphorylation level of hallmark proteins in fetal heart at late-gestation day 135; **(O–U)**. Relative expression and phosphorylation level of hallmark proteins in neonatal heart at day 1 after birth; GAPDH, Protein loading control. CON, control, MO, maternal obesity, M, male, and F, female. Mean ± SEM (*n* = 5); *p < 0.05, **p < 0.01.

Autophagy is an effective internal regulatory mechanism that allows biological organisms to adapt to different environments and protect organisms from metabolic stress (Levine and Kroemer, 2008). Either the enhancement or the suppression of autophagy is observed in obesity (Ignacio-Souza et al., 2014). Whether MO induces or suppresses autophagy flux in offspring tissues remains unclear. Studies have linked MAPK signaling with autophagy (Zhou et al., 2015; Zhang et al., 2018). To estimate alteration of autophagy associated signaling in heart tissue of fetuses and neonates of obese ewes, the family members involved in MAPK signaling, such as extracellular signal-regulated kinase (ERK)1/2, JNK, and p38 MAPK, were selected as hallmark proteins to be examined using western blotting. We first detected the protein expression of hallmark proteins at mid-gestation day 90 (GD90) in control and MO male and female fetal sheep hearts. The results showed that total ERK expression in control females was increased compared with control males (*p* = 0.0125). MO female fetuses also had increased total ERK expression compared with MO male fetuses (*p* = 0.0058) (Figures 1A,B). However, phosphorylation of ERK was not altered between groups (*p* > 0.08) except that pERK2 in MO female fetuses was increased compared with MO male fetuses (*p* = 0.0140) (Figures 1A,C). JNK and p38 MAPK expression were not different between all groups (*p* > 0.09) (Figures 1A,D,F), however, the phosphorylation of JNK was increased in only control Females and the phosphorylation level of p38 MAPK in MO heart was higher by comparing to their respective control in both genders (Male *p* = 0.0306; Female *p* = 0.0038) (Figures 1A,E,G). At GD 135, total ERK had no difference between treatment group and control group (Male *p* = 0.1573; Female *p* = 0.4874). Sex differences were observed between control groups. Total ERK was increased in control females compared with control male (*p* = 0.0125) (Figures 1H,I). Phosphorylation of ERK had no changes between all groups (*p* > 0.05) (Figures 1H,J). Total JNK expression was reduced in MO female fetuses compared to control female fetuses (*p* = 0.0071), but not in MO male fetuses vs Control male fetuses (*p* > 0.05) (Figures 1H,K). However, JNK expression was significantly different in control females compared with control males (*p* = 0.0149) (Figures 1H,K). Phosphorylation ratio of JNK had no differences between each group in GD 135 fetal heart (*p* > 0.05) (Figures 1H,L). Both P38 MAPK expression and phosphorylation had no differences in MO vs control in male fetuses (*p* > 0.05), but were lower in MO female fetuses compared with control female fetuses (Expression *p* = 0.0033 and Phosphorylation *p* = 0.0014), and the female control was higher than that in male control (Expression *p* = 0.0385 and phosphorylation *p* = 0.0092) (Figures 1M,N). At day1 after birth, we found that total ERK was increased in MO male neonates compared with control male neonates (*p* = 0.0154) and MO female neonates (*p* = 0.0051) respectively (Figure 1O,P). Phosphorylation of ERK2 was increased in MO female neonates compared with control female (*p* = 0.0064) and MO male neonates (*p* = 0.0105) respectively (Figure 1Q). JNK was increased in MO female neonates compared to control female neonates (*p* = 0.0427) and reduced in control female neonates compared to control male neonates (*p* = 0.0014) (Figure 1R). Phosphorylation of JNK was not significantly different between each group (*p* > 0.05) (Figure 1S). No differences were found for the expression and phosphorylation of P38 MAPK between each group (*p* > 0.05) (Figure 1T,U).

### Activation of the PI3K/AKT/mTOR Signaling Pathway in Overfed Male and Female Fetal and Neonatal Heart

**FIGURE 2:**
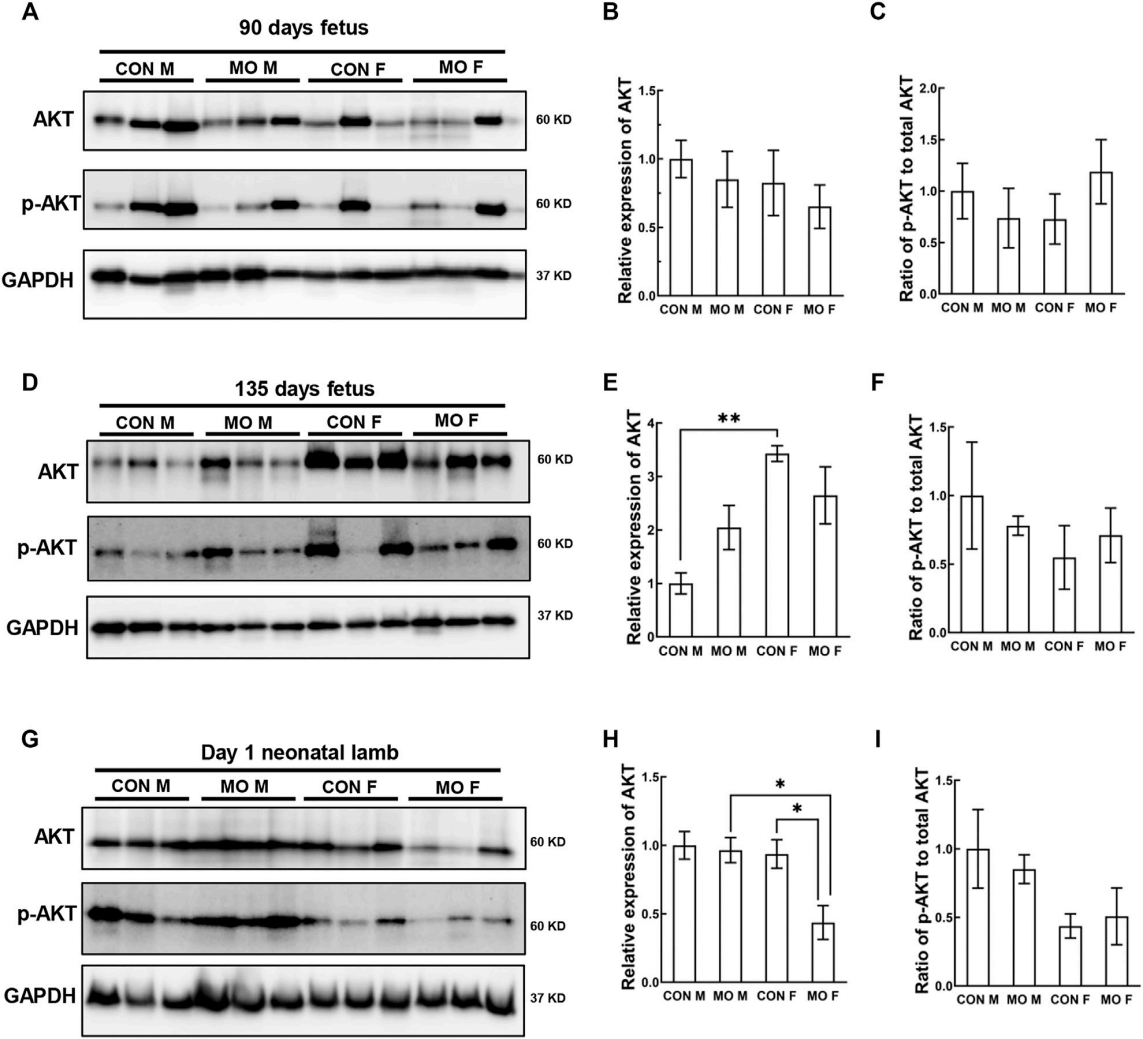
Alteration of AKT signaling pathway in offspring heart of overfed ewes at two gestational and one early postnatal time point. **(A–C)**. Relative expression and phosphorylation level of the kinase Akt in fetal heart at mid-gestation day 90; **(D–F)**. Relative expression and phosphorylation level of the kinase Akt in fetal heart at late-gestation day 135; **(G–I)**. Relative expression and phosphorylation level of the kinase Akt in neonatal heart at day 1 after birth; GAPDH, Protein loading control. CON, control, MO, maternal obesity, M, male, and F, female. Mean ± SEM (*n* = 5); *p < 0.05, **p < 0.01.

The kinase mammalian target of rapamycin (mTOR) is a major regulator of the autophagic process which is indirectly regulated by the survival PI3K/AKT pathway, the upstream of mTOR (Heras-Sandoval et al., 2014). We then detected expression level and activity of Akt in our overfed sheep model at DG90, DG135, and day 1 after birth. At DG90, we did not observe significant changes of Akt expression and phosphorylation level in MO groups compared with control groups nor in sex comparisons (*p* > 0.05) (Figures 2A–C). At DG135, Akt expression was only increased in control females compared with control males (*p* = 0.0028), suggesting the protective role in female relative to male in response to maternal stress (Salminen et al., 2012; Ricke-Hoch et al., 2014). However, there was no difference between other treated and untreated groups (*p* > 0.05) (Figures 2D–F). At day 1 after birth, we observed decreased expression of Akt in MO female neonates compared with control female (*p* = 0.0202) and MO male neonates (*p* = 0.0153) respectively, and no differences were observed between any other groups (*p* > 0.05) (Figures 2G–I).

### Expression of the Autophagy-Related Proteins (ATGs) in Overfed Male and Female Fetal and Neonatal Hearts

**FIGURE 3:**
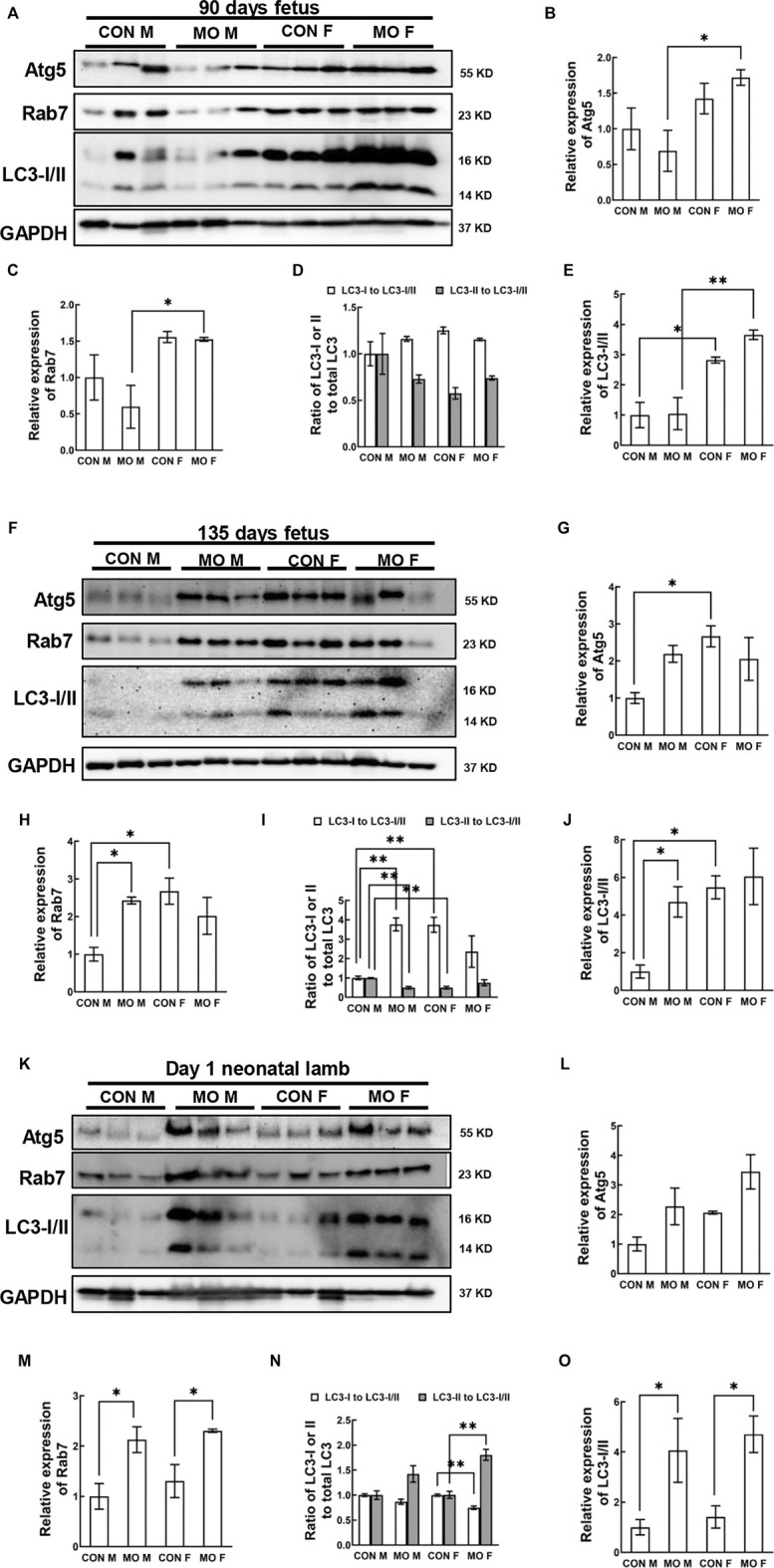
Alteration of autophagy signaling pathway in offspring heart of overfed ewes at two gestational and one early postnatal time point. **(A–E)**. Relative expression and phosphorylation level of hallmark proteins in fetal heart at mid-gestation day 90; **(F–J)**. Relative expression and phosphorylation level of hallmark proteins in fetal heart at late-gestation day 135; **(K–O)**. Relative expression and phosphorylation level of hallmark proteins in neonatal heart at day 1 after birth; GAPDH, Protein loading control. CON, control, MO, maternal obesity, M, male, and F, female. Mean ± SEM (*n* = 5); *p < 0.05, **p < 0.01.

Autophagy is regulated by a number of signaling molecules, particularly the ATG family (Kim and Lee, 2014; Galluzzi et al., 2014;Galluzzi et al., 2017). Atg5 is considered an important ATG, as it is indispensable in both canonical and non-canonical autophagy (Bouderlique et al., 2016; Ye et al., 2018). Microtubule-associated protein 2 light chain 3 (LC3-II) and GTPase Rab7 are other common markers of autophagy in mammals (Tanida et al., 2004; González-Rodríguez et al., 2014; Kjos et al., 2017; Zheng et al., 2019). Because of their importance in the autophagy process and availability of antibodies that cross-react in sheep, we chose these autophagic hallmarks to indicate the changes of autophagic flux in the offspring of MO ewes. At DG90, we found that there were no differences in Atg5, Rab7, and LC3-I/II between MO and control fetuses (*p* > 0.05), however, sex differences were observed (Atg5 of MO group *p* = 0.0313; Rab7 of MO group *p* = 0.0335; LC3-I/II of control group *p* = 0.0126; LC3-I/II of MO group *p* = 0.0015) (Figures 3A–E). Atg5 (*p* = 0.0313) and Rab7 (*p* = 0.0335) were increased in MO female fetuses compared with MO male fetuses (Figures 3B,C); Total LC3-I/II was increased in females compared with males between controls (*p* = 0.0126) and between MO fetuses (*p* = 0.0015) respectively (Figure 3E). At DG135, control female fetuses had the increased ATG5 (*p* = 0.0195), and Rab7 (*p* = 0.0115) when compared to control male fetuses (Figures 3F–H). The ratio of LC3-I to II was decreased in MO male fetuses compared to control male fetuses (*p* = 0.0074) and the decreased ratio was also observed in control female fetuses compared with control male fetuses (*p* = 0.0077) (Figures 3F,I). Total LC3-I/II was increased in MO male fetuses (*p* = 0.0437) and control female fetuses (*p* = 0.0178) compared to control male fetuses (Figure 3J). At day 1 after birth, Atg5 level had no differences between any groups (*p* > 0.05) (Figures 3K,L). Rab7 was increased in MO male neonates compared to control male neonates (*p* = 0.0233) and in MO female neonates compared with control female neonates (*p* = 0.0404) (Figures 3K,M). The ratio of LC3-I to II was increased in MO female neonates compared to control female neonates (*p* = 0.0021) (Figures 3K,N). Total LC3-I/II was increased in MO male neonates compared with control male neonates (*p* = 0.0482) and in MO female neonates compared with control female neonates (*p* = 0.0350) (Figures 3K,O).

### Cortisol Concentration in Overfed Male and Female Fetal and Neonatal Heart

**FIGURE 4:**
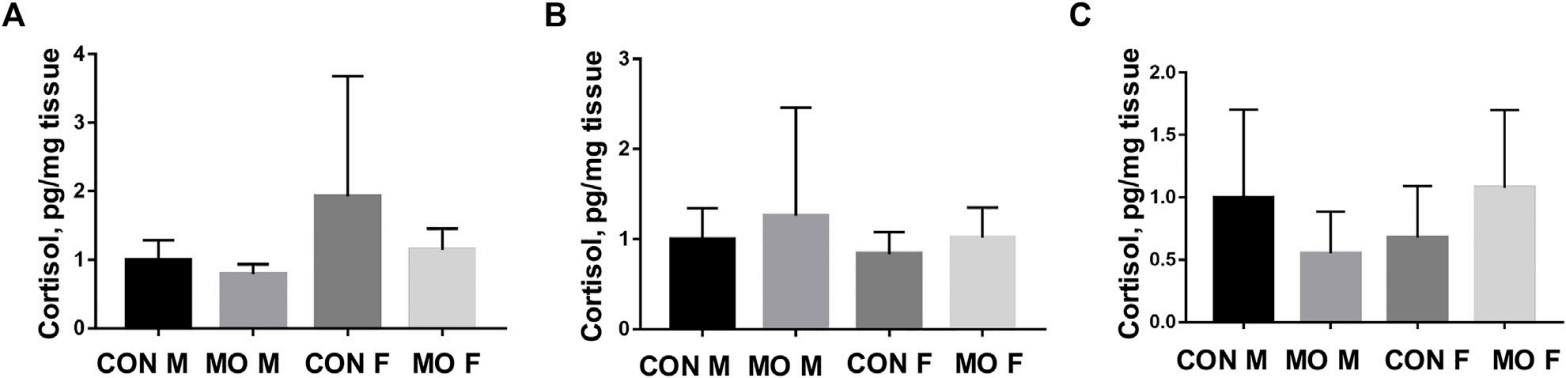
Cortisol concentration in heart tissues of offspring of overfed ewes at two gestational and one early postnatal time point. **(A)** Cortisol concentration in fetal heart tissues at day 90 of gestation; **(B)** Cortisol concentration in fetal heart tissues at day 135 of gestation; **(C)** Cortisol concentration in neonatal heart tissues at day 1 after birth; CON, control, MO, maternal obesity, M, male, and F, female. Mean ± SEM (*n* = 5). No significance.

Studies have shown that cortisol concentrations are elevated in MO mothers and fetuses at both mid- and late-gestation (Buss et al., 2012; Nathanielsz et al., 2013; Odhiambo et al., 2020). Growing evidence has suggested that excessive cortisol is associated with autophagy (Swerdlow et al., 2008; Harr et al., 2010; Ahn et al., 2014; Wnuk and Kajta, 2017). To evaluate whether cortisol concentrations are increased in MO fetal and neonatal hearts, we performed ELISA assays to detect cortisol concentrations at DG90, DG135, and day 1 after birth. The results indicated that there were no changes observed in the heart of overfed fetuses and neonatal sheep at three developmental stages (at DG90, *p* = 0.436; at DG135, *p* = 0.491 and at day 1, *p* = 0.697) (Figures 4A–C).

### Expression of Inflammatory Cytokines in Overfed Male and Female Fetal and Neonatal Hearts

**FIGURE 5:**
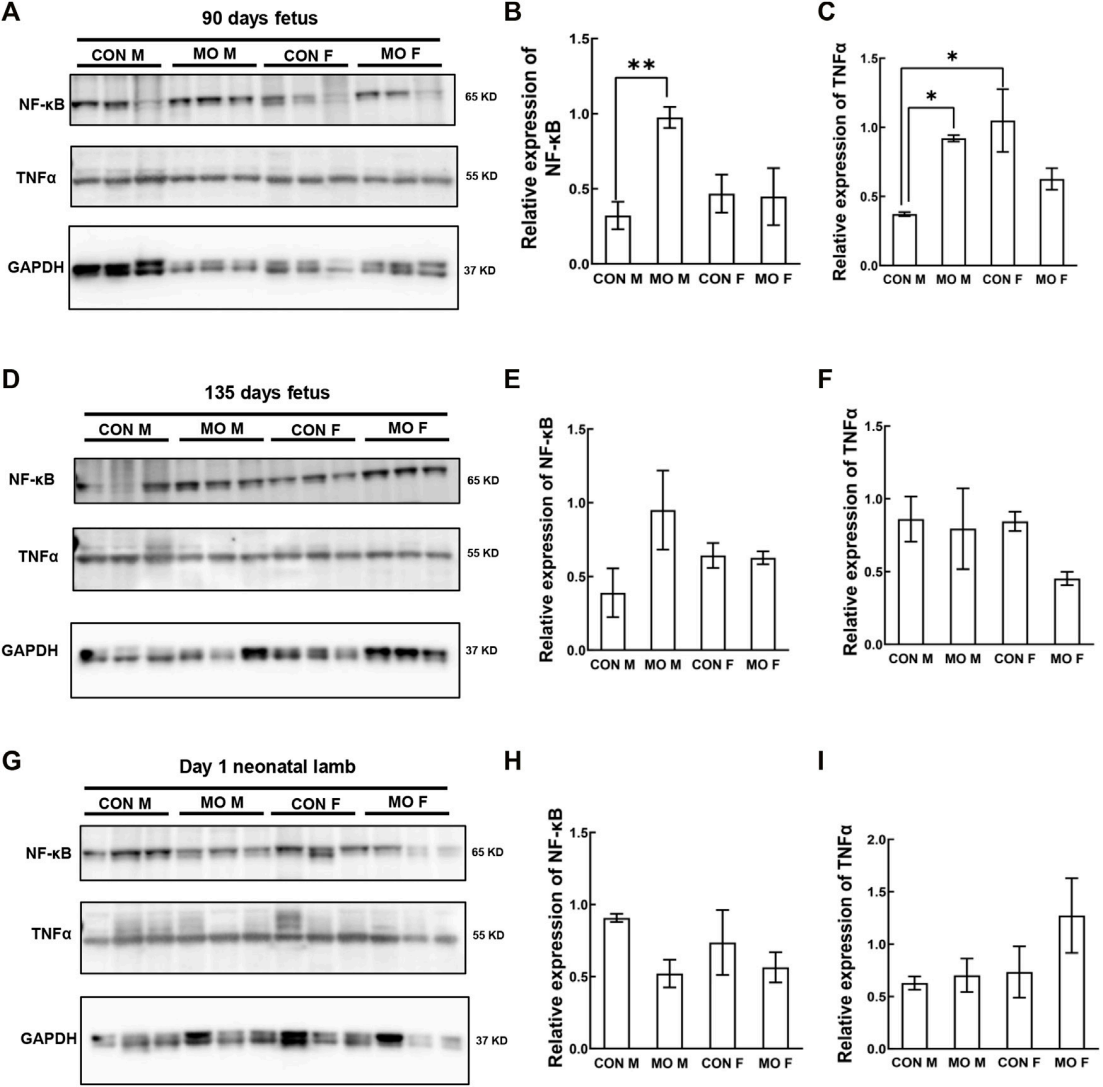
Alteration of inflammatory cytokine NF-κB and TNFα in offspring heart of overfed ewes at two gestational and one early postnatal time point. **(A–C)**. Relative expression level of inflammatory cytokine in fetal heart at mid-gestation day 90; **(D–F)**. Relative expression level of inflammatory cytokine in fetal heart at late-gestation day 135; **(G–I)**. Relative expression level of inflammatory cytokine in neonatal heart at day 1 after birth; GAPDH, Protein loading control. CON, control, MO, maternal obesity, M, male, and F, female. Mean ± SEM (*n* = 5); *p < 0.05, **p < 0.01.

Autophagy can also be regulated by inflammation (Qian et al., 2017; Matsuzawa-Ishimoto et al., 2018). To further explore whether MO-induced autophagy in later developmental stage is associated with inflammation, the expression of inflammatory cytokines NF-κB and TNFα was determined. At DG90, the expression of NK-κB was significantly higher in MO male fetuses than that in control (*p* = 0.0075), but no difference was detected between MO, and control female fetuses (*p* > 0.05) (Figures 5A,B). The expression of TNFα in control male fetuses was significantly lower than that in MO male (*p* = 0.0263) and control female fetuses (*p* = 0.0084) (Figures 5A,C). However, the expression of both inflammatory cytokines was not different between each group in DG 135 fetuses and neonates (*p* > 0.05) (Figures 5D–I).

## Discussion

Poor maternal nutrition during gestation can alter lamb growth rates, tissue composition, and organ size at early postnatal time points (Godfrey and Barker, 2001; Hoffman et al., 2014; Reed et al., 2014; Hoffman et al., 2016; Raja et al., 2016). Our previous study showed that MO impairs fetal heart contractile function (Wang et al., 2019), which may be associated with changes in autophagy. However, little is known about the effects of poor maternal nutrition on heart muscle in the overfed sheep model. In this study, we further evaluated whether overfeeding during gestation affects autophagy associated pathways at two gestational and one early postnatal time point in male and female offspring. As expected, we observed altered expression of autophagy associated proteins in MAPK/ERK signaling, PI3K/AKT/mTOR signaling and autophagy signaling pathways in both male and female offspring between MO and controls at late gestational stage (GD135) and early postnatal stage (day 1 after birth). Most intriguingly, our results suggest altered autophagy associated pathways exhibit sex differences. We found that female offspring were more profoundly influenced than male offspring by overfeeding of the dam during gestation.

**FIGURE 6:**
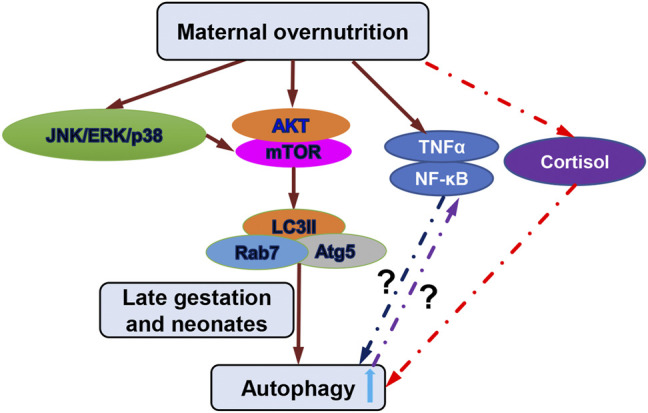
Schematic diagram of autophagy associated signaling pathways and potential mechanisms regulated by maternal overnutrition. Important hallmark proteins in the PI3K/Akt/mTOR and the MAPK/ERK signaling are indicated which were examined in this study. Alternation of these hallmark proteins in later gestation and early postnatal life increased autophagy. Cortisol level did not change in heart tissues and was not the cause for the increased autophagy in later gestation and early postnatal life indicated by red dash line. Inflammatory factors TNFα and NF-κB were altered by maternal overnutrition in early gestation but not late gestation or early postnatal life in which the possible mechanisms could be that increased inflammatory factors in early gestation inhibits autophagy (blue dash line and question mark) and unchanged inflammatory factors at late gestation and early postnatal life could be suppressed by increased autophagy (purple dash line and question mark).

Autophagy is a conserved process that catabolizes unnecessary and/or dysfunctional intracellular components for quality control to attenuate stress and maintain cellular homeostasis (Cheng et al., 2013; Sinha et al., 2017). The autophagic process is regulated by a number of signaling molecules, among which the mTOR kinase has a master role (Figure 6; Galluzzi et al., 2014; Galluzzi et al., 2017). The kinase mTOR induces phosphorylation of the autophagy-initiating ULK1 molecular complex and suppresses formation of autophagosome and autophagolysosome through a number of autophagy-related proteins including ATG5, LC3, and Rab7 (Figure 6; Ganley et al., 2009; Hosokawa et al., 2009; Jung et al., 2009). mTOR is a downstream target of the kinases PI3K and AKT (Manning and Cantley, 2007; Heras-Sandoval et al., 2014). Activated AKT can phosphorylate and activate mTOR and thus suppress autophagy through ULK1/2 (Figure 6; Inoki et al., 2002; Saucedo et al., 2003; Menon et al., 2014). In addition, MAPKs, in particular p38 MAPK, activate mTOR in autophagy signaling. Furthermore, ERK and p38 MAPK regulate autophagy in response to various stimuli (Figure 6) (Webber, 2010). Recent studies show that JNK, one of MAPK subfamily members is also involved in the regulation of autophagy in response to environmental stress (Zhou et al., 2015). Examination of these signaling pathways that indirectly regulate autophagy through mTOR in overfed fetal and neonatal hearts demonstrated that overnutrition alters signaling that regulates autophagy in offspring of obese ewes (Figure 6). However, the effects are more overt in late gestation and early postnatal life. For example, at GD90, the autophagy associated proteins Atg5, Rab7, and LC3-I/II were not activated in overfed male or female offspring; however, at GD135 and day 1 after birth, these proteins were activated, suggesting altered autophagy, in both males and females. A handful of studies have shown that autophagy level is upregulated in response to extra- or intracellular stress signals such as starvation, nutrient signaling, energy balance, and stress signaling etc. (He and Klionsky, 2009). Maternal obesity in sheep may lead to increased food intakes, low birth weight and energy balance regulation in late gestation and early postnatal life (Muhlhausler et al., 2006) which could explain why we observed the elevated autophagy pathway in late gestation and early postnatal life. Although the overall situation is more complicated, our data show that MO is a risk factor that may activate autophagy processes indirectly through upstream signaling of mTOR kinases in offspring heart muscles.

On the other hand, glucocorticoids have been linked to the induction of autophagy in response to stress in the cell such as nutrient deprivation (Juhász et al., 2003; Kinch et al., 2003; Kuma et al., 2004; Levine and Klionsky, 2004; Martin et al., 2007). Studies using under- and over-nutrition sheep model demonstrated that cortisol level was elevated in fetuses at the mid-term gestation and late-term gestation as well as in newborn lambs (Dong et al., 2008; Ford et al., 2009; Long et al., 2011; Zhang et al., 2011; Smith et al., 2018; Odhiambo et al., 2020). These studies suggest that autophagy associated signaling pathways could be induced by elevated cortisol level in fetal heart and neonatal hearts. However, our results revealed that MO fetal and neonatal hearts did not increase the cortisol level, implying that other potential mechanisms such as inflammation, may induce the altered autophagy associated protein expression. Inflammation is a common outcome of MO in many models (Moreli et al., 2012; Tarry-Adkins et al., 2016; Ghnenis et al., 2017; Jones et al., 2018; Montalvo-Martínez et al., 2018). Inflammation can regulate autophagy in both positive and negative ways (Trocoli and Djavaheri-Mergny, 2011; Salminen et al., 2012; Shi et al., 2016; Wu et al., 2016). We have tested the expression of two inflammatory factors TNFα and NF-κB in fetal and neonatal heart. The results showed that, compared to the control, the protein level of NK-κB and TNFα were all upregulated in GD90 MO male fetuses, but not in fetuses of later gestation stage (GD135) or neonates. Studies in sheep showed that TNFα or TNF super family member 11 were upregulated in the late gestation stage (GD135) or postnatal lambs (22-month-old) of overfed or obese ewes (Ghnenis et al., 2017). However, reports also showed that inflammation was not increased in fetal rat tissue in late gestation exposing to maternal obesity (Crew et al., 2016). Inflammatory response may vary in different organ and at different age of the offspring. In the present study, the sensitivity of inflammation to MO was opposite to that of autophagy at different developmental stages. Previous studies showed opposite trend of autophagy and inflammation in kidney (Nguyen et al., 2017; Sato et al., 2019), adipose tissue (Jansen et al., 2012), and placenta (Upadhyay et al., 2019) of offspring in response to maternal malnutrition. Considering the complex crosstalk between autophagy and inflammation (Harris et al., 2017; Deretic and Klionsky, 2018; Matsuzawa-Ishimoto et al., 2018), the possible mechanisms could be that 1) the inflammatory cytokines may inhibit the autophagy in early gestation stage, but were depressed by other factors in later gestation stage and after birth; or 2) the autophagy raised in the later stage inhibited the expression of inflammatory cytokines. The precise mechanisms by which the autophagy at the late stage of MO fetuses was induced in our study remain further investigation.

Lastly, effects of MO on the regulation of autophagy in other tissues or organs has been studied in animal models and human placenta. Studies using rodent models have reported that autophagy markers were suppressed in the kidneys of offspring of obese mothers with sex specificity (Nguyen et al., 2017). In a study with human placenta and mouse model, the researchers reported a sexual dimorphism in placental autophagy in response to MO (Muralimanoharan et al., 2016). Recent study in a MO sheep model demonstrated that autophagy protein markers were not altered in late-term MO F1 fetal livers (Serafim et al., 2021). The inconsistencies of altered and unaltered autophagy protein markers in different animal models and human studies indicate assessment of changes in autophagy with obesity can be rather complicated, as they depend on the nature, duration and models of obesity used, the tissue or cell types tested or simply the autophagy monitoring techniques used (Klionsky et al., 2016; Soussi et al., 2016; Zhang et al., 2018). Therefore, although our study supports that MO alters markers of autophagy in offspring hearts of obese ewes, future studies are required to determine if autophagy itself is altered and if these results can be transmitted to future generations.

## Data Availability

The original contributions presented in the study are included in the article/Supplementary Material, further inquiries can be directed to the corresponding author.
